# Biocompatible artificial synapses based on a zein active layer obtained from maize for neuromorphic computing

**DOI:** 10.1038/s41598-021-00076-1

**Published:** 2021-10-19

**Authors:** Youngjin Kim, Chul Hyeon Park, Jun Seop An, Seung-Hye Choi, Tae Whan Kim

**Affiliations:** 1 Department of Electronic Engineering, Hanyang University, Seoul, 04763 Republic of Korea; 2 Center for Neuroscience, Brain Science Institute, Korea Institute of Science and Technology (KIST), Seoul, 02792 Republic of Korea

**Keywords:** Engineering, Electrical and electronic engineering

## Abstract

Artificial synaptic devices based on natural organic materials are becoming the most desirable for extending their fields of applications to include wearable and implantable devices due to their biocompatibility, flexibility, lightweight, and scalability. Herein, we proposed a zein material, extracted from natural maize, as an active layer in an artificial synapse. The synaptic device exhibited notable digital-data storage and analog data processing capabilities. Remarkably, the zein-based synaptic device achieved recognition accuracy of up to 87% and exhibited clear digit-classification results on the learning and inference test. Moreover, the recognition accuracy of the zein-based artificial synapse was maintained within a difference of less than 2%, regardless of mechanically stressed conditions. We believe that this work will be an important asset toward the realization of wearable and implantable devices utilizing artificial synapses.

## Introduction

In the emerging Big Data era, represented by artificial intelligence (AI) and the Internet of Things (IoT), it is facing the challenge of handling large numbers of formal datasets, as well as unstructured data formats, such as images, characters, and sounds^1–3^. Under these technical circumstances, neuromorphic computing, which depends on parallel and nonlinear data processing and mimics the biological human brain, has become a subject of tremendous interest^4–6^, and neuromorphic computing using conventional von Neumann structures based on the complementary metal–oxide–semiconductor (CMOS) circuit system has been achieved^7,8^. However, the system not only requires a large number of CMOS transistors to implement the neuron and synapse circuits but also faces significant problems, such as high power consumption because the computing is based on centralized and sequential processing with a clock cycle^9–11^. Therefore, new neuromorphic computing devices with high energy efficiency and high density needs to be developed.

The memristor, which is a portmanteau of memory and resistor, has a dynamic relationship between voltage and current based on past currents or voltages applied to it^12^. Memristor devices can gradually change their conductance according to input pulses from past currents, and they have been shown to have the ability to emulate the brain's synaptic plasticity, as well as its functions, such as learning, memory, and cognition, because of their capability to modulate their conductances^6,13–16^. Therefore, memristive devices have been actively studied as artificial synaptic devices^13,15^. Resistance switching random access memory (ReRAM)-based memristive devices are promising due to their advantages of reliability, low power consumption, and fast operation^17–19^. In particular, the ReRAM-based artificial synaptic device can implement synaptic functions within a unit device and can be constructed with high density via fabrication in a crossbar array structure due to its simple two-terminal structure (metal–insulator-metal) ^20–22^.

Natural organic materials are promising candidates for use as components of artificial synapses due to their superior advantages, such as pliability, low-temperature processability, scalability, lightweight, and compatibility with various substrates^13,23–25^. Particularly, their biocompatibility allows the extension of their applications to advanced implantable, wearable, bio-integrated nanoelectronic devices^26,27^. Recently, ReRAM and memristive devices based on natural materials have been reported, but the application of such materials to artificial synaptic devices is rarely reported^28–30^. In addition, although organic materials originate from nature, they may still be toxic to the human body after the fabrication step. Therefore, proving biocompatibility after fabrication is essential; however, this is rarely done.

In this study, we demonstrated the feasibility of using a zein-based memristor as a bio-compatible artificial synaptic device. Zein, which is extracted from natural maize, was applied as the active layer between the top and the bottom electrodes. The biocompatibility of zein was investigated by using cellular toxicity tests on normal human skin cells. Then, the digital data storage and the analog data processing capabilities of the zein-based artificial synaptic device were characterized. Based on changes in the device ‘s conductance behavior and the use of a conventional learning algorithm in a single-layer neural network, we investigated the recognition capability of our zein-based artificial synapse. Finally, to evaluate the possibility of using our zein-based device for wearable and implantable nanoelectronics, we tested its mechanical stability during digital and analog data processes.

## Results and discussion

**Figure 1:**
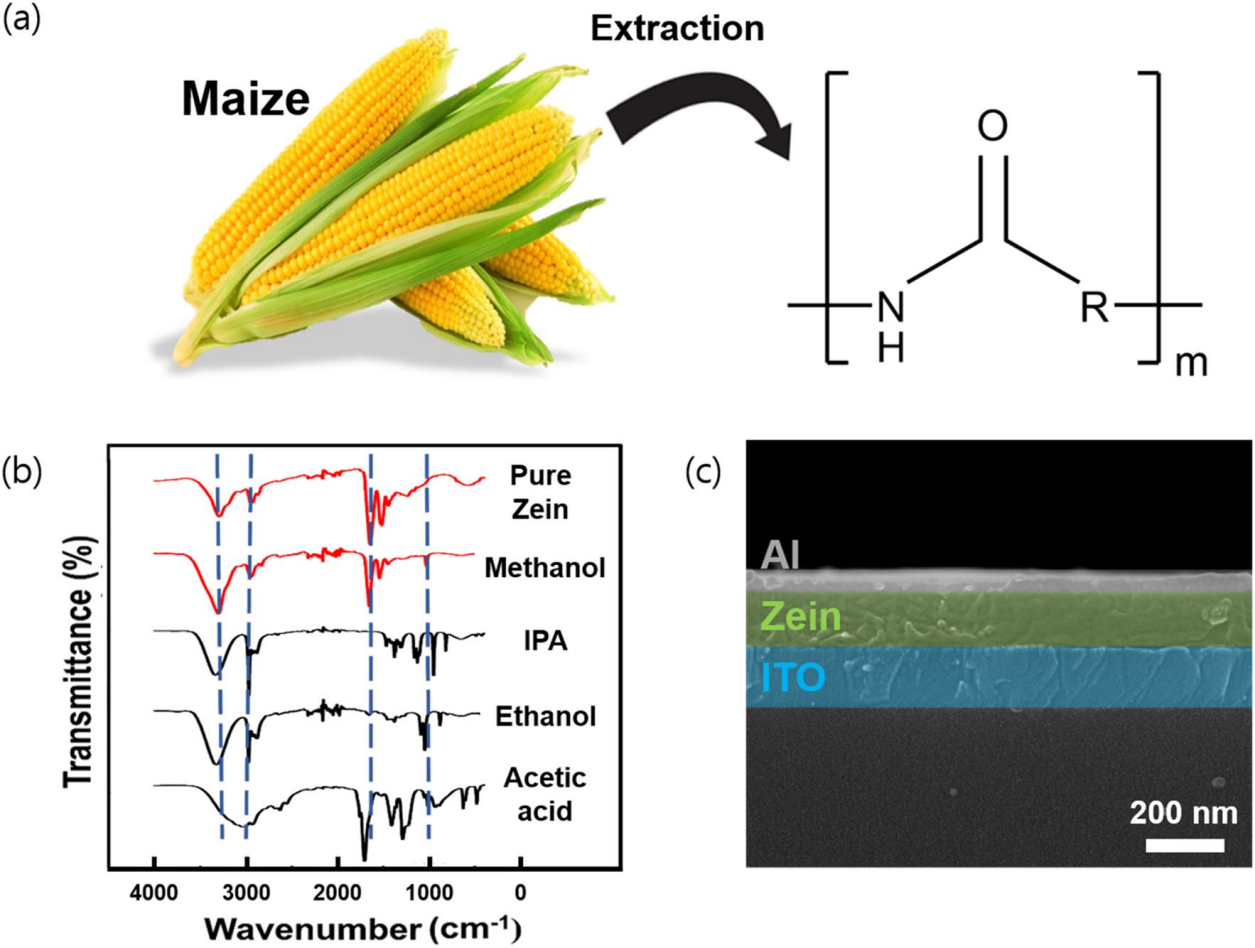
(**a**) Optical images of the maize and the zein powders, and chemical structure of zein. (**b**) FT-IR spectra for the pure zein and the zein films prepared using the indicated solvents. (**c**) Cross-sectional SEM image of the Al/zein/ITO structure.

Zein is extracted from natural maize, and its molecular structure is given in Fig. 1a. In general, the amino acid composition of the zein includes glutamic acid, leucine, proline, alanine, phenylalanine, isoleucine, serine, and tyrosine^31^. Zein can be classified as α-, β-, γ-, or δ-zein base on its solubility and its amino acid sequence homology, and about 80% of the total mass of zein is α-zein. Because of the amino acids in zein, such as glutamine, leucine, and proline, it can dissolve in a limited number of solvents, including alkaline solutions and aqueous alcohols^32^. Figure 1b shows the Fourier-transform infrared spectroscopy (FT-IR) results for pure zein powder and for zein films prepared by dissolving it in methanol, isopropyl alcohol, ethanol, and acetic acid, respectively. Each solvent was selected based on the solubility of zein in the solvent and its accessibility. In all the spectra, major transmittance peaks were observed at wavenumbers at of 3300, 2950, 1650, and 1250 cm^−1^ corresponding to O–H, C–H, C=C, and C–O bonds, respectively^33^. As a result of comparing the FT-IR spectra of pure zein with those from the zein films prepared by using various solvents, we were able to confirm that the zein film could be prepared from zein in methanol without specific chemical decomposition. Figure 1c shows the SEM result for a zein film fabricated on an ITO glass substrate. This result demonstrates that a zein layer with a thickness of about 185 nm had been successfully deposited on the substrate.

**Figure 2:**
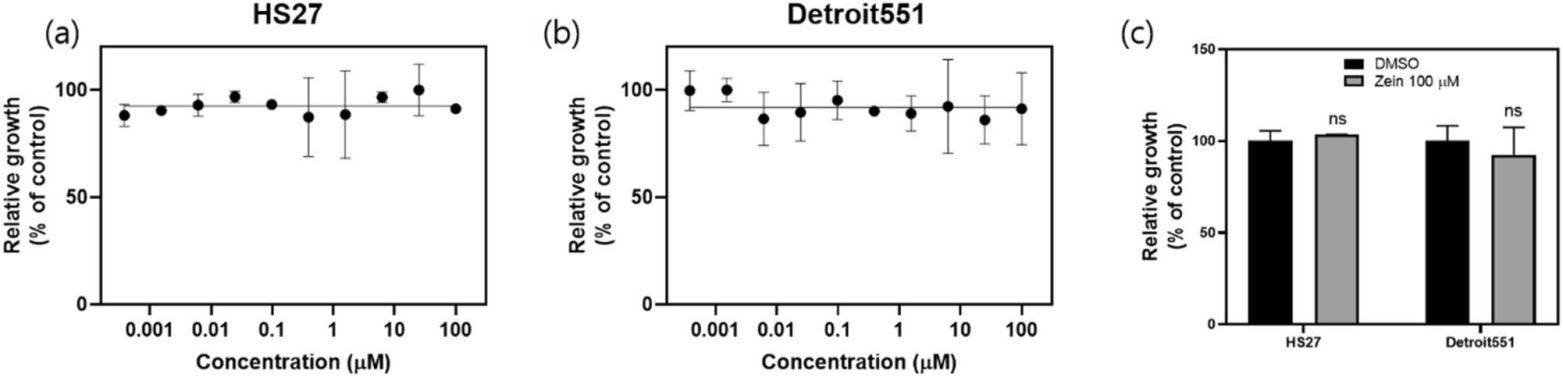
Biocompatibility results for zein and anti-proliferative abilities of zein organics for normal (**a**) HS27 and (**b**) Detroit551 skin cells. Note that the CellTiter-Glo assay demonstrated up to a 100-μM concentration of zein after 72 h of cell growth. (**c**) The relative growth results for normal HS27 and Detroit551 skin cells in the DMSO solvent and in zein at a 100-μM concentration after 72 h.

As we mentioned in the introduction, the main reason for developing zein-based devices is to confirm the possibility of developing human-friendly devices that can be applied as wearable or smart healthcare devices. In these applications, the devices may be attached to the human skin and even inserted inside the human body. To confirm the cytotoxicity of zein, therefore, we dissolved zein in a methanol solvent with a concentration of 1 wt%. The zein was deposited on a glass substrate by spin coating at 2000 rpm for 40 s. The film was thermally baked for 30 min to remove solvent residues. After that, the zein film was detached from the glass substrate. The anti-proliferative ability of the film was measured using its dose- and time-dependent effects on normal HS 27 (fibroblast, male) and Detroit 551 (fibroblast, female) skin cells. For the dose-dependent effect, the concentration of zein in dimethyl sulfoxide (DMSO) was adjusted from 0.001 to 100 μM, and for the time-dependent effect, measurements were made for up to 72 h. CellTiter-Glo assays demonstrated that at least up to a concentration of 100 μM, zein did not affect the viability of either the HS 27 or the Detroit 551 cells over a period of 72 h, as shown in Fig. 2 and Fig. S1. In addition, by comparison with the control test for DMSO, which is the base solvent, we were able to confirm that zein did not affect the viability of either the HS 27 or the Detroit 551 skin cells (Fig. 2c). These results indicate that zein is highly biocompatible.

**Figure 3:**
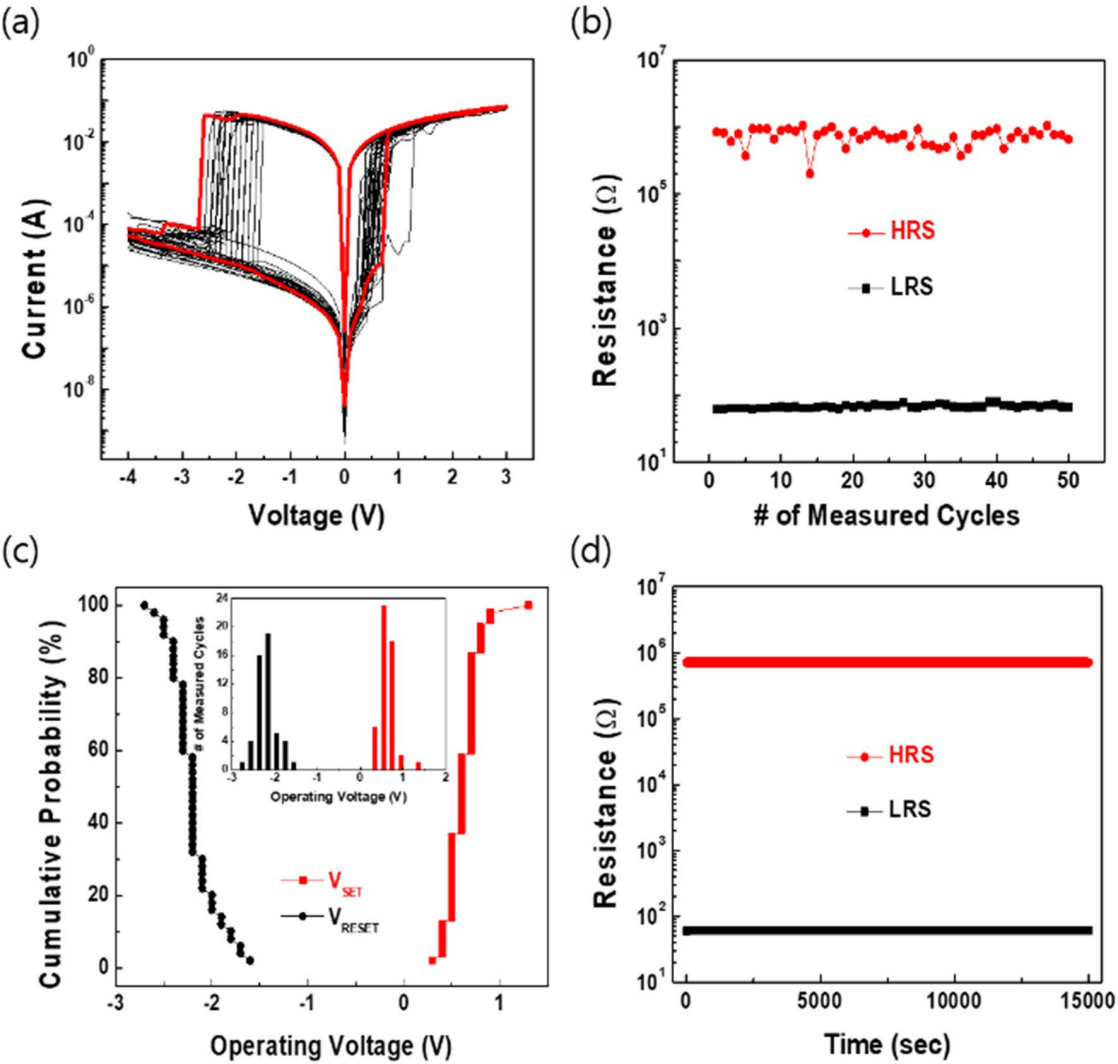
(**a**) Current–voltage curves on a semi-logarithmic scale for the Al/zein/ITO device for 50 cycles. The thick red line shows the result after the first sweep. (**b**) Endurance performance in the DC sweep mode for 50 cycles. (**c**) Cumulative probabilities for the SET and the RESET voltages in the DC sweep mode. (**d**) Retention test result over 10^4^ s at 295 K.

The digital characteristics of the zein-based device were evaluated by sweeping the voltage applied to the device in the order −4 → 0 → 3 → 0 → −4 V under a compliance current of 0.1 A and a 0.1-V/s sweep rate. In general, the thickness of the active layer is a critical factor for memristive performance based on conductive filaments. Thus, the I–V characteristics of devices with active zein layers of different thicknesses, which could be controlled by changing the spin-coating speed, were evaluated (Fig. S2). The device with an active zein layer fabricated at a spin-coating speed of 2000 rpm showed stable resistance switching operation with low SET/RESET voltages and a high R_ON_/R_OFF_ ratio. The result for the 1st electrical sweep of the device prepared with spin-coating speed of 2000 rpm is indicated by the thick red line in Fig. 3a, which shows a typical bipolar resistance switching (BRS) behavior. The resistance state of the device is switched from a high resistance state (HRS) to a low resistance state (LRS) at 0.7 V in the positive voltage region. Then, the LRS of the device remained until the voltage reached 0 V again. When the voltage sweep was conducted in the negative voltage region, the LRS returned to the HRS at − 2.6 V. After the first electrical sweep, when subsequent I–V sweeps were carried out, for up to 50 cycles, these resistance switching behaviors successfully repeated without any failure or degradation of the operation parameters, including the SET (i.e., resistance switching from HRS to LRS) and the RESET (i.e., resistance switching from LRS to HRS) voltages, as well as the R_ON_/R_OFF_ ratio. The distributions of the SET and the RESET voltages for 50 repeated operations are summarized in Fig. 3b. The average values and the standard deviations of the SET and the RESET voltages were 0.62 ± 0.16 and − 2.20  ± 0.23 V, respectively, demonstrating notably uniformity in the switching behavior. The resistance values and their standard deviations for the LRS and the HRS were 67.31 ± 4.14 Ω and 7.29 × 10^5^ ± 1.87 × 10^5^ Ω (Fig. 3c), respectively, and the R_ON_/R_OFF_ ratio in the endurance test was relatively constant at 4 × 10^4^. These results obviously confirm the stable switching operation of the zein-based device. The retention result is shown in Fig. 3d. Both programmed resistance states, the HRS and the LRS, showed no degradation at a 0.2 V of read voltage for over 10^4^ s. In the programmed pulse switching test, the zein-based device exhibited fast and reproducible resistance-switching operation for up to ~ 10^4^ switching cycles, as shown in Fig. S3. For the programmed pulse conditions, the SET and the RESET pulse heights (length) were 1.3 V (200 ns) and − 3 V (250 ns), respectively. During the pulse switching test, digital switching between the SET and the RESET behaviors was repeated without failure. In addition, the frequency dependence of the corresponding capacitance in the frequency range from 100 kHz to 1 MHz at a 0.2 AC voltage was determined (Fig. S4), and the capacitive states of our zein-based memristive device were found to have a capacitance of about 60 fF, which varied very little with frequency.

**Figure 4:**
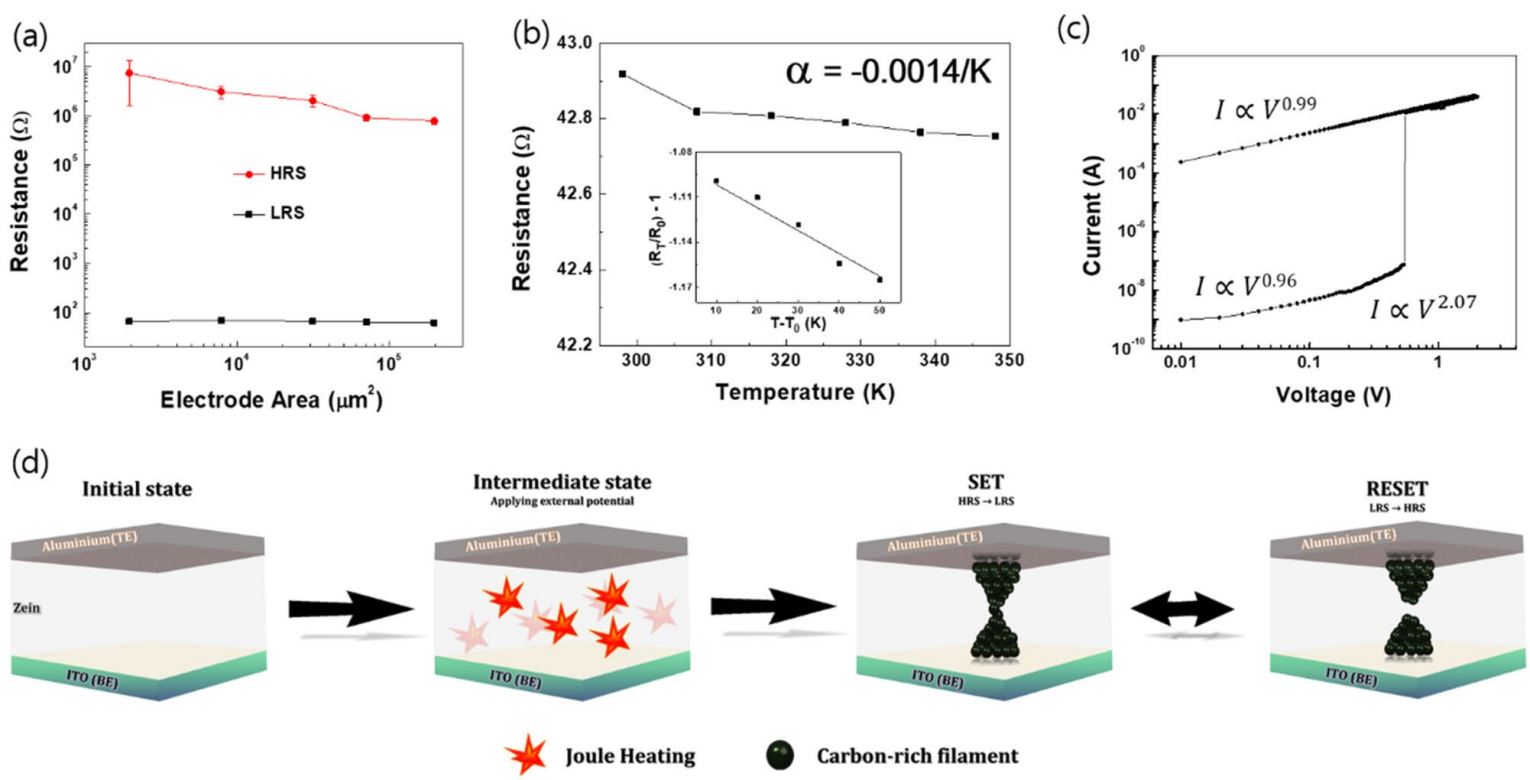
(**a**) Electrode-area dependence of the zein-based device. (**b**) Variation in the resistance with temperature for the zein-based device. The inset shows the temperature coefficient of resistance in the LRS. (**c**) Double logarithmic plot in a positive sweep region from the high resistance state to the low resistance state. (**d**) Schematic illustration of the switching mechanism for the device with the Al/zein/ITO structure. When an external bias is applied to the device, pyrolysis in the zein active layer begins due to Joule heating. Consequently, a conductive carbon-rich filament is formed between the Al top electrode and the ITO bottom electrode. The carbon-rich filament can be ruptured by thermal driving due to an applied negative bias.

The results so far indicate that the zein-based device holds promise for applications in a ReRAM device. To identify the switching mechanism of the zein-based device for meaningful resistance switching results, we examined the area-dependent resistance switchings for Al top electrodes with different diameters from 50 μm to 500 μm. The corresponding resistance values of the LRS and the HRS are shown in Fig. 4a and Fig. S5a as functions of the cell area. With increasing electrode area, the resistance of the HRS exhibited a certain dependency whereas the resistance of the LRS showed an obviously slight variability, compared to the HRS, as a function of the area of the electrode, implying that the switching behaviors of our zein-based device are clearly caused by the filamentary resistance switching mechanism^34,35^. To determine the origin of the filaments in a zein insulating medium, we examined the temperature dependence of the resistance for the Al/zein/ITO device. Figure 4b and Fig. S5b show the variation in the resistance of the LRS for increasing temperature from 298.15 K to 348.15 K; a decrease in the resistance of the LRS with increasing temperature is observed. To clarify this issue, we evaluated the temperature coefficient of resistance for the zein-based device by using the following equation^36^:

1 $$R_{T} = R_{o} \left[ {1 + \alpha \left( {T - T_{o} } \right)} \right]$$

where *α*, *R*_*T*_, and *R*_*o*_ are the temperature coefficient of resistance, the resistance of the LRS at the temperature *T*, and the resistance of the LRS at the initial temperature *T*_*o*_, respectively. *α* is calculated to be − 0.0014/K through a linear fitting of the data by using the inset of Fig. 4b, which is comparable to the reported value for polycrystal carbon (− 0.0011/K)^36^. These results, obtained from the temperature dependence tests, imply that the zein-based device operates through the construction of a carbon-based conductive filament (CF) in a zein insulating medium when an external bias is applied to the zein layer. The conduction mechanism of the zein-based device was determined by considering double-logarithmic plots of the I–V curves in Fig. 3a. As shown in Fig. 4c, the fitted curve in the HRS region exhibits two different slopes, one for low (0–0.27 V) voltage and the other for high (over 0.27 V) voltage. In the low voltage region, the slope is approximately 1, indicating that the current transport is dominated by Ohm’s law. This results from the electric field between the two electrodes being insufficient, meaning that the number of injected charge carriers is lower than the number of thermally generated free charge carriers. At voltages above 0.27 V, the slope of the curve increases to approximately 2, indicating that the Mott-Gurney law and Child’s law govern the current transportation via the space charge limited current (SCLC) mechanism^37^. In the LRS region, the fitted curve indicates that Ohmic conduction is dominant.

Considering the results so far, we propose a schematic for the resistance switching mechanism based on the presence of carbon-rich CFs in the Al/zein/ITO device, as shown in Fig. 4d. The proposed resistance switching mechanism is derived from the construction of carbon-rich CFs caused by a local change in the zein structure, which is independent of the two electrodes. In this suggested resistance switching model, the construction of carbon-rich CFs can be explained via the pyrolysis of the zein layer due to Joule heating. When an external voltage is applied to the device, Joule heating can be triggered in the zein medium, leading to the pyrolysis of the zein polymer^38–40^. As the applied voltage is increased (i.e., accelerated pyrolysis), the localized carbon-rich region expands, and eventually, the resistance state of the zein-based device is changed from the HRS to the LRS when both electrodes are connected by a localized carbon-rich region. After the construction of the carbon-rich CFs, the RESET process can occur through ruptures at the weak points in the localized carbon-rich CFs due to a combination of the reverse reaction and Joule heating. Studies related to localized carbon-rich filaments have found that the resistance switching caused by carbon-rich filaments is a BRS behavior; moreover, no electroforming step, which is a characteristic behavior in electrochemical metallization, was observed^40^. These characteristics are exactly the same as the resistance switching properties of the zein-based device.

**Figure 5:**
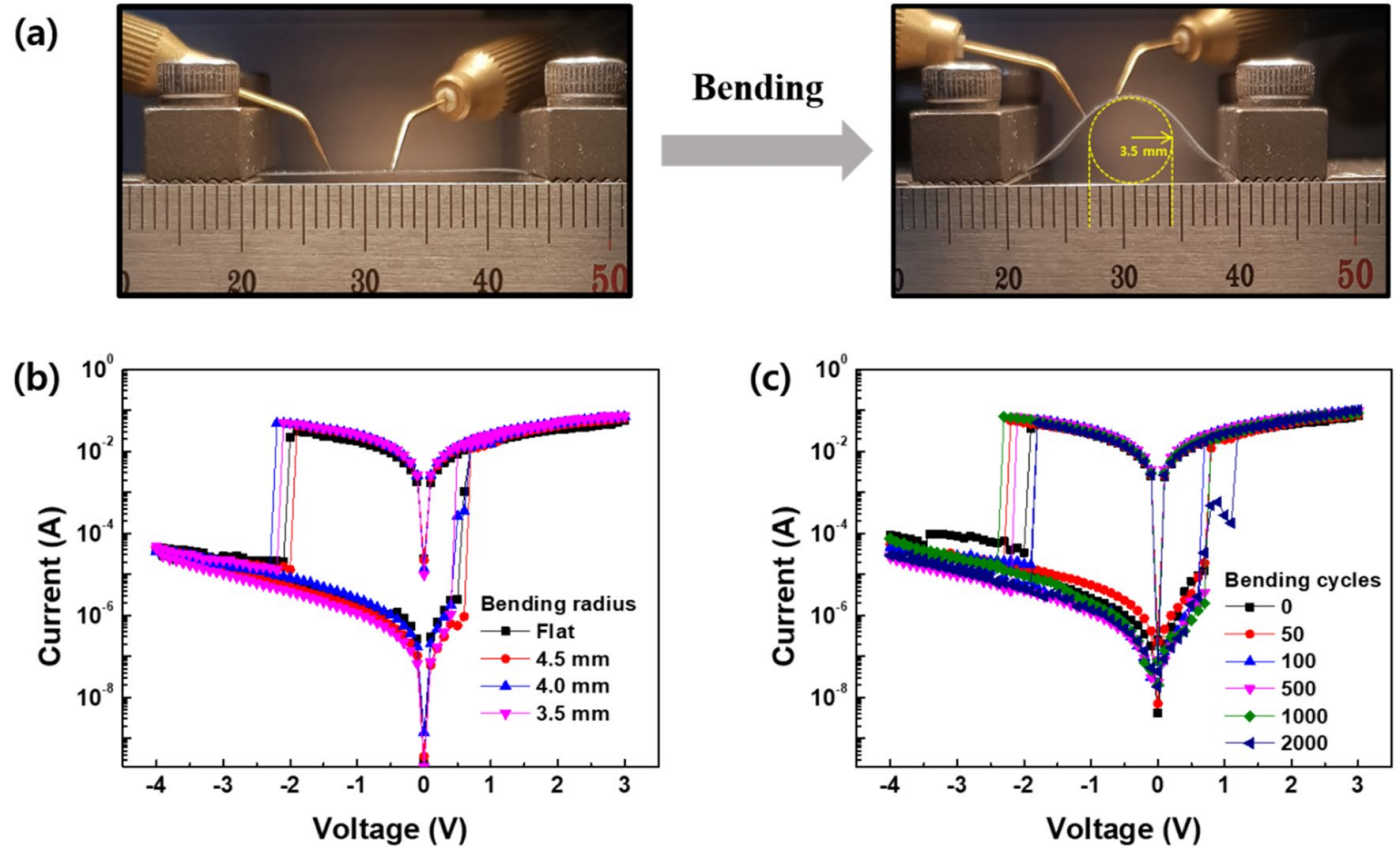
(**a**) Photographs of the zein-based device subjected to bending from the flat state. (**b**) Comparison of I–V curves under mechanical deformation from the flat state to 3.5 mm of bending radius and (**c**) bending cycles up to ~ 2000 cycles.

As mentioned in the introduction, one possible application of the zein-based device is as flexible or wearable electronics. To demonstrate the flexibility of the zein-based device, we cast the zein layer on an ITO/polyethylene naphthalate (PEN) substrate. Then, the Al top electrode was deposited on the zein film. The deformation-dependent resistance switching performance of the device was evaluated by adjusting the bending radius from a flat state to 3.5 mm, as shown in Fig. 5a. The *I–V* characteristics, including operation voltages and R_ON_/R_OFF_ ratios, of the zein-based device during bending were almost the same as those of the flat device, even under the maximum bending condition of 3.5 mm (Fig. 5b). In addition, when the bending and unbending was conducted for up to ~ 2000 cycles, the initial resistance switching behavior was maintained without any degradation, as shown in Fig. 5c. This notable operation stability under mechanical stress is due to the inherent elasticity of the zein resistive-switchable layer.

**Figure 6:**
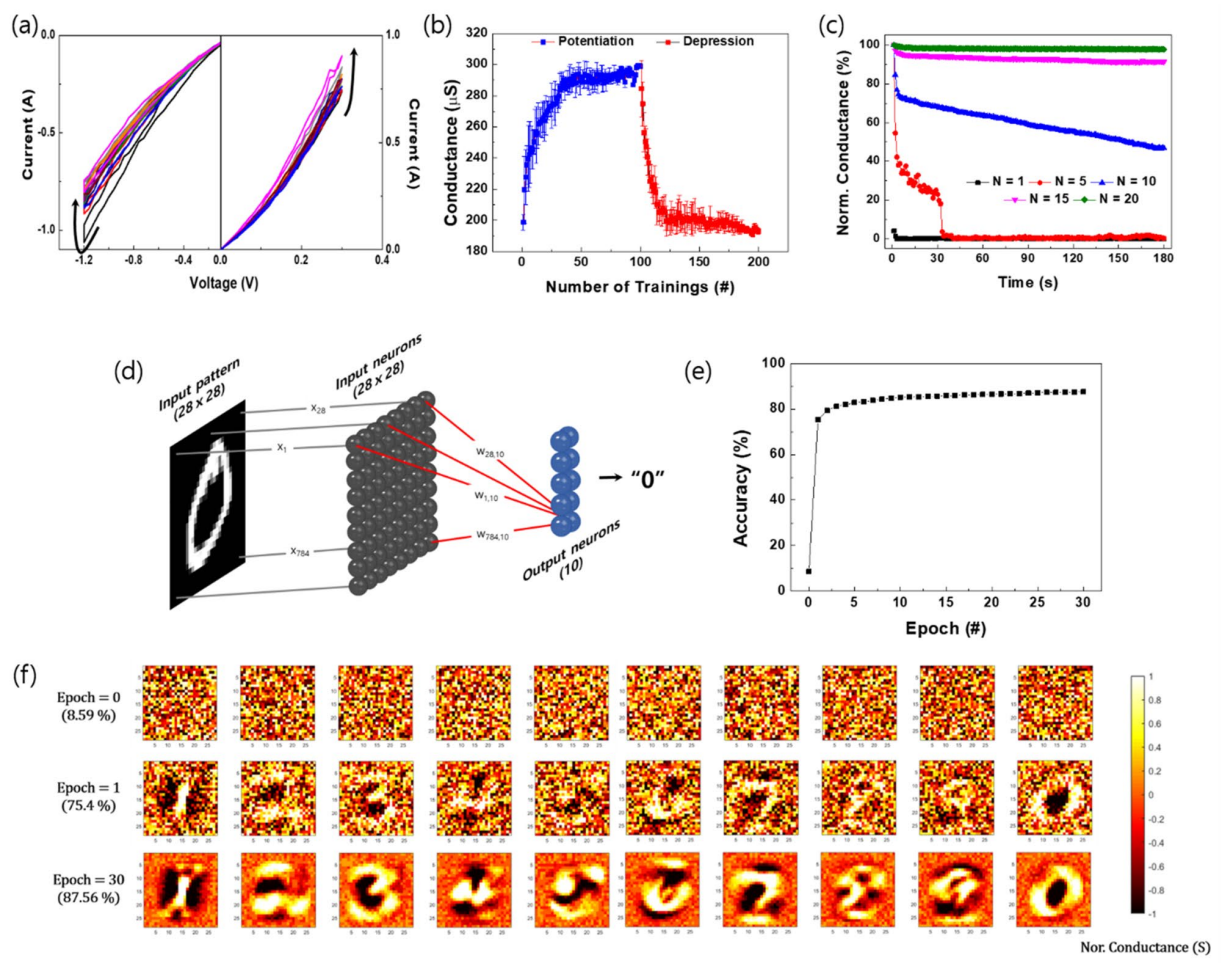
(**a**) Potentiation and depression characteristics for 10 cycles in the direct current sweep mode. (**b**) Average potentiation and depression results over 10 consecutive cycles. (**c**) Characteristics of the memory transition from STM to LTM caused by the programming pulses (N = 1, 5, 10, 15, and 20). (**d**) Schematic diagram of a single-layer network for the “0” pattern recognition process. The input pattern “0” (28 × 28), input neurons (28 × 28, gray), and output neurons (2 × 5, blue) are fully connected. (**e**) Recognition accuracy as a function of the number of epochs (learning phases). (**f**) Weight-mapping images at zero, the 1st, and the 30th training epoch.

If the complex neurological functions of the human brain is to be realized in a memristive device, emulating the variations in the synaptic weight (i.e., conductance in the case of a memristive device) is the most important factor. Here, we investigated the biomimetic neurological properties of the zein-based artificial synaptic device, and the results are shown in Fig. 6. Initially, the current gradually increased and decreased over each DC sweep, as shown in Fig. 6a. To avoid any abrupt change in the current, we used a total of 10 consecutive sweep cycles (0 V → 0.3 V → 0 V) and 10 reverse sweep cycles (0 V → −1.2 V → 0 V), where the voltages were smaller than the threshold voltage. When the positive sweep was first applied, the current at 0.3 V was 0.74 μA and then increased to 0.91 μA in the 10th sweep. Subsequently, when the negative sweep was first applied, − 0.86 μA was measured at − 1.2 V, and then changed to − 0.75 μA in the 10th negative sweep. Obviously, the analog behavior of our zein-based synaptic device can be explained by the cumulative operation history related to the construction of the carbon-rich CFs via a Joule heating-related mechanism. For potentiation, 100 programmed paired pulses, which consisted of a 0.2-V read voltage, and a 0.4-V training pulse with 1 μs, were applied to the device. Next, the depression process was evaluated 100 times (0.2-V read voltage, and − 1.8-V training pulse with 1 μs). The conductance change was plotted by calculating an average for 10 consecutive potentiation and depression processes, and the results are shown in Fig. 6b. When the first training was applied to the device, the conductance was measured as 197.8 ± 5.1 μS, but after 100 consecutive potentiation trainings, the conductance was increased to 299.1 ± 1.2 μS. This analog behavior means that the training of the applied positive pulses is a potentiation process. Subsequently, when the paired pulses for the depression process were applied, the conductance value decreased continuously. After the 100th depression process, the conductance had almost returned to its initial value (192.9 ± 1.9 μS). When we calculated the energy consumption for potentiation/depression of the zein-based memristive device, we found that the average energy consumptions for potentiation and depression were 0.08 μW/event and 0.41 μW/event, respectively, which suggests that our device has a highly energy-efficient memristive behavior. The potentiation curve is monotonically smaller than the depression curve because this behavior is derived from the construction of carbon-rich CFs due to Joule heating. In general, because Joule heating is based on a current produced by an applied voltage, at larger voltages, more drastic changes can occur during the depression process than during the potentiation process. In addition, as the number of potentiation and depression trainings was increased, the accuracy of the conductance improved, as shown in Fig. S6. Humans obtain knowledge via learning and reconstruction by remembering^41^. Memory is defined as short-term memory (STM) or long-term memory (LTM): memory that disappears within a minute is STM while memory that lasts over minutes is LTM. In general, memory can be converted from STM to LTM by manipulating rehearsals. A programming pulse (N) was applied to our zein-based device; then, the relaxation of the conductance was traced for 180 s. Figure 6c shows the behavior of the conductance relaxations after having applied programming pulses (N = 1, 5, 15, and 20) to the device. When up to five programming pulses were applied to the device, the conductances rapidly returned, within a minute, to their initial values. However, the decay rates of conductances decreased with increasing programming pulse. The normalized conductance values for N = 1, 5, 10, 15, and 20 after 180 s were 46.9, 91.4, and 97.8%, respectively. Therefore, when more that 10 programming pulses are applied, the memory state of the device can be regarded as LTM.These results showing an analog conductance changeable behavior caused by potentiation and depression processes in the zein-based artificial synaptic device clearly indicate that our device can imitate biological synaptic-plasticity.

Nowadays, a synaptic device is a promising component in neuromorphic computing for digit or face recognition with high power efficiency and is expected to solve the problems of a machine-learning system based on Von Neumann architecture. To evaluate the recognition capability of our zein-based synaptic device, we simulated, based on the fitting result for the potentiation and the depression processes in Fig. 6a,b pattern classification task by using the MNIST (Modified National Institute of Standards and Technology). The details of the MNIST simulation and the learning algorithm are summarized in Supplementary Information section. The pattern recognition process is illustrated in Fig. 6d. Seven hundred eighty-four (784) input patterns (28 × 28 input pixels) are fully connected with input neurons (28 × 28 input neurons) and 10 output neurons (2 × 5 output neurons). Thus, 7840 pairs of an individual synapse have their synaptic weight, and 10 output neurons correspond to the numbers from "0" to "9". As shown in Fig. 6e, the recognition accuracy without a training epoch initially was 8.59%. After the 1^st^ training epoch, the recognition rate was improved to 75.43%, and a recognition rate of 87.59% was achieved after the 30th epoch. When the mapping images of 784 synaptic weights corresponding to the digits from “0” to “9” were visualized for the 0th, 1st, and 30th training epochs (see Fig. 6f), the mapping images after the 30th training iteration were recognized more clearly than those after the 0th and the 1st training epochs (see Fig. S7).

**Figure 7:**
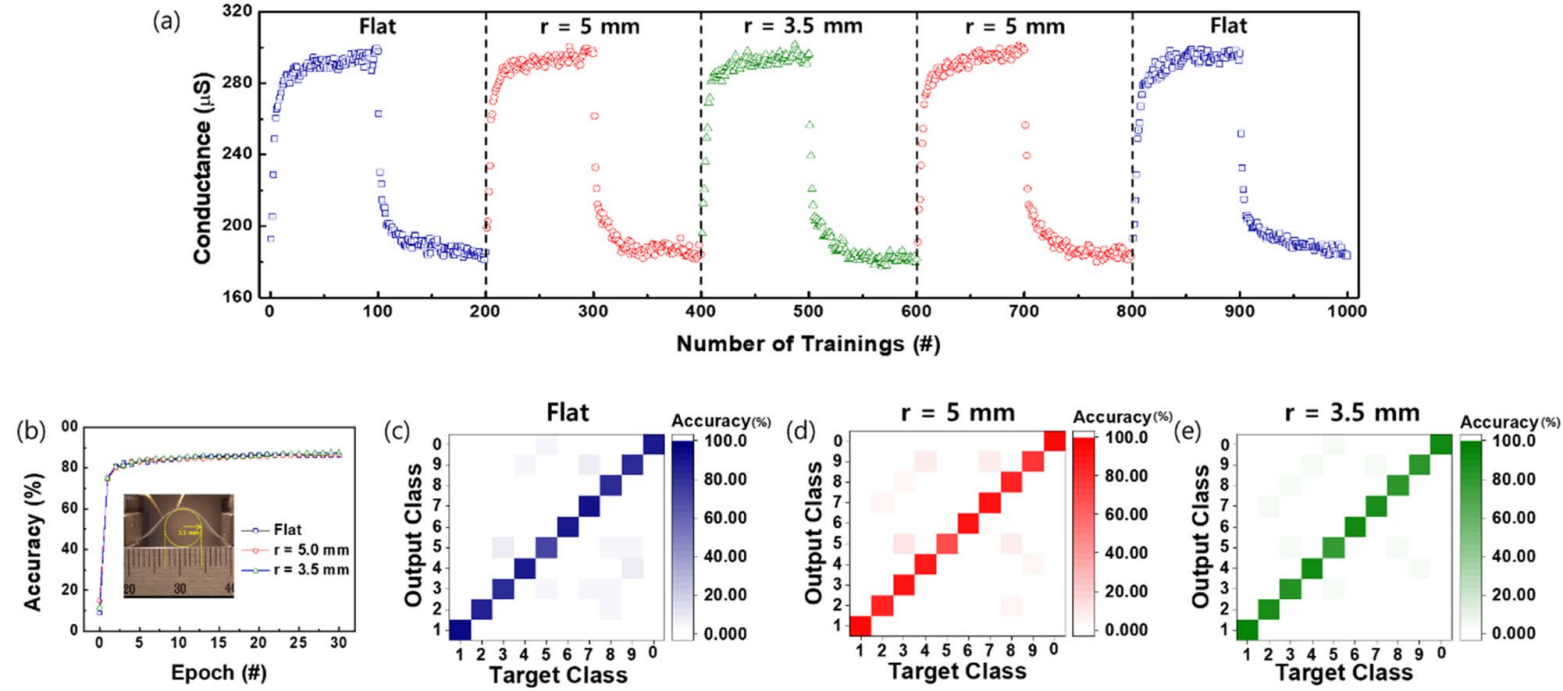
(**a**) Potentiation and depression characteristics corresponding to consecutive pulse trainings against mechanical stress from a flat state to 3.5-mm bending state. (**b**) Comparison of the recognition accuracies under different mechanical stresses. (**c**)-(**e**) Classifications for the learning and the inference test in different bending states [(**c**) flat, (**d**) r = 5 mm, and (**e**) r = 3.5 mm]. Note that the classifications were visualized by using confusion matrices (10 × 10) between the target classes (input digit) and the output classes (learning phases).

To confirm the mechanical stability of the machine-learning capability in our zein-based synaptic device, we applied the programmed paired pulses for potentiation and depression to devices with different fixed bending radii of 0 mm (flat), 4.5 mm, and 3.5 mm. Each potentiation and depression cycle under the mechanical-stress condition of bending exhibited very little changes compared with the cycle in the flat state, as shown in Fig. 7a. Thus, the variations in the recognition accuracies of the sample for 0 mm, 4.5 mm, and 3.5 mm bending were less than 2% after the 30th training epoch (Fig. 7b). Learning and inference tests based on the above results were conducted by using 60,000 and 10,000 different MNIST patterns. The results from the bending tests were concretely visualized by the confusion matrix (10 × 10), meaning the classification results between the targeted class (input pattern) and the output MNIST patterns (Fig. 7c-e). The tiles with the saturated royal color in the matrix of Fig. 7c based on the potentiation and the depression results without bending were arranged diagonally, meaning that each digit was correctly inferred by learning. As shown in Figs. 7d and e, the tiles in the confusion matrices were all arranged diagonally with the greatest saturated color, as expected. These results mean that the zein-based synaptic device can accurately infer and recognize patterns, regardless of mechanical-stress conditions.

## Conclusions

In conclusion, an artificial synaptic device based on zein, which can be extracted from maize, was successfully fabricated. Firstly, the cytotoxicity test proved that the zein-based synapse was human-friendly. Secondly, the zein-base artificial synapse was demonstrated to have well-defined digital data storage and analog data processing capabilities. In more detail, the device exhibited excellent resistive switching behaviors with reliable operating parameters: 0.62 ± 0.16 V at SET and − 2.2  ± 0.23 V at RESET, with a large R_ON_/R_OFF_ ratio of 4 × 10^4^. Furthermore, the zein-based artificial synapse was shown to be able to imitate biological synaptic-plasticity through its changeable analog conductance behavior. On the basis of the meaningful results of our research, notably, that the zein-based device achieved recognition accuracy of about 87% in the MNIST simulation, we were able to demonstrate that the digits from "0" to "9" could be correctly inferred by learning. Particularly, the superior digital and analog capabilities of the zein-based synapse were maintained within a difference of less than 2%, regardless of the fixed bending conditions, thereby enabling accurate inference and recognition. We believe that these results will lay a critical foundation for the further development of biocompatible neuromorphic electronic devices.

## Methods

### Device fabrication

Artificial synaptic devices with a structure of Al/zein/ITO were fabricated. The zein was purchased from Sigma-Aldrich Corporation (CAS NO 9010-66-6). ITO-coated glass was ultrasonically cleaned using acetone, ethanol, and distilled water for 15 min each and then dried under blowing N_2_ gas. Zein was dissolved in various solvents, such as methanol, ethanol, isopropanol, and acetic acid, at a concentration of 1 wt%. Each solution was stirred for over 24 h under ambient conditions. The zein active layers were deposited on the ITO glass substrates by spin coating at 2000 rpm for 40 s. Then, the films were thermally baked at 50 °C for 30 min to remove solvent residues. Finally, an Al top electrode with a 250-μm-diameter and 40-nm-thickness was deposited by using thermal evaporation at a pressure of 3 × 10^6^ Torr.

### Biocompatibility test

HS27 and Detroit551 cells purchased from ATCC and KCLB (Seoul, Korea) were cultured in Dulbecco's modified eagle medium (DMEM) supplemented with 10% (v/v) fetal bovine serum (FBS), penicillin (100 μg/mL), and streptomycin (100 μg/mL) in a humidified 5% CO_2_ incubator at 37 °C. For the cellular toxicity test, 5.0 × 10^3^ cells per well were seeded in a 96-well plate, and the plate was stored overnight, after which the test compounds were added to the wells in a 1:4 serial dilution in dimethyl sulfoxide (DMSO) up to a concentration of 100 μM. After 72 h, the cellular viability was determined by using Cell Titer-Glo reagent (G7572, Promega, USA). A dose–response curve was fitted, and the GI_50_ values were calculated using Graphpad Prism 6.0 software. All assays were performed in triplicate, and the standard deviation (S.D.) was determined from three independent experiments.

### Characterization

The digital and the analog properties of the zein-based artificial synapse were analyzed using a Keithley 4200 semiconductor characterization system (SCS) containing two source measure units and pulse measure units (Keithley 4225-PMU) in a probe station. The morphological characterization was done using a field-emission scanning electron microscope (FE-SEM, JEOL JSM-7100F) operating at 15 kV. The structural characteristics of the formed zein active layers were investigated using Fourier-transform infrared spectroscopy (FT-IT, NICOLET iS50).

### MNIST simulation

The detailed methods are described in Supplementary Information.

## Supplementary Information

Supplementary Information.
