# Supplementary material for: Biocompatible artificial synapses based on a zein active layer obtained from maize for neuromorphic computing
Source: Sci Rep. 2021 Oct 19;11:20633. doi: 10.1038/s41598-021-00076-1 (PMC8526676; doi:10.1038/s41598-021-00076-1)
Supplement: Supplementary file 1 — Supplementary Information. [file 41598_2021_76_MOESM1_ESM.docx]

Supplementary Information

Biocompatible artificial synapses based on a zein active layer obtained from maize for neuromorphic computing

Youngjin Kim,^1,#^ Chul Hyeon Park,^1,#^ Jun Seop An,^1^ Seung-Hye Choi,^2^ Tae Whan Kim^1,^*

^1 Department of Electronic Engineering, Hanyang University, Seoul 04763, Republic of Korea^

^2 Center for Neuroscience, Brain Science Institute, Korea Institute of Science and Technology (KIST), Seoul 02792, Korea^

*Corresponding authors: Prof. T. W. Kim ([twk@hanyang.ac.kr](mailto:twk@hanyang.ac.kr))

^#^Y. Kim and C. H. Park contributed equally to this work.


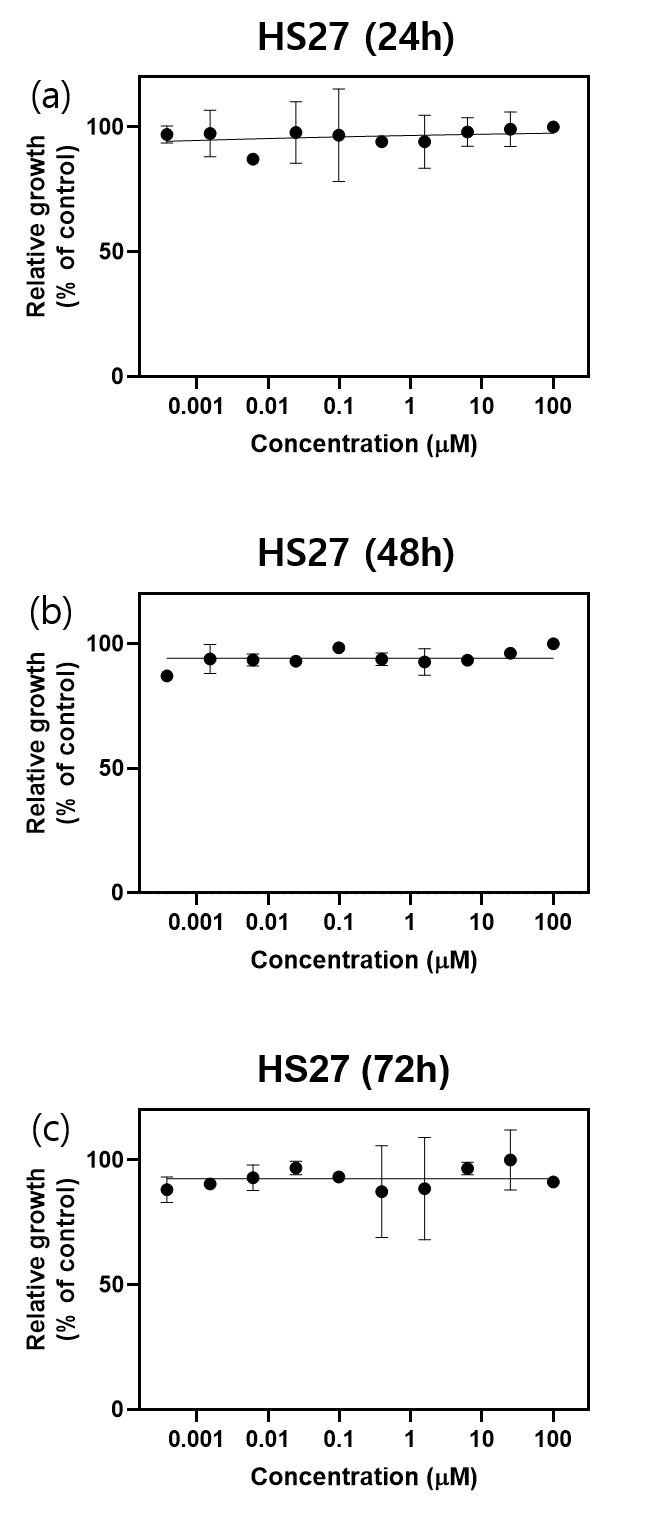


**Figure S1.** Results of biocompatibility tests on the zein for up to 72 h.

**Figure S2.** The I-V characteristics of the devices prepared by different spin-coating speeds.

**Figure S3.** Pulse switching behavior of the Al/zein/ITO device for up to ~10^4^ switching cycles. [SET and RESET pulse heights (length) are 1.3 V (200 ns) and -3 V (250 ns), respectively.]

**Figure S4.** Frequency dependence of capacitance of the zein-based memristive device.


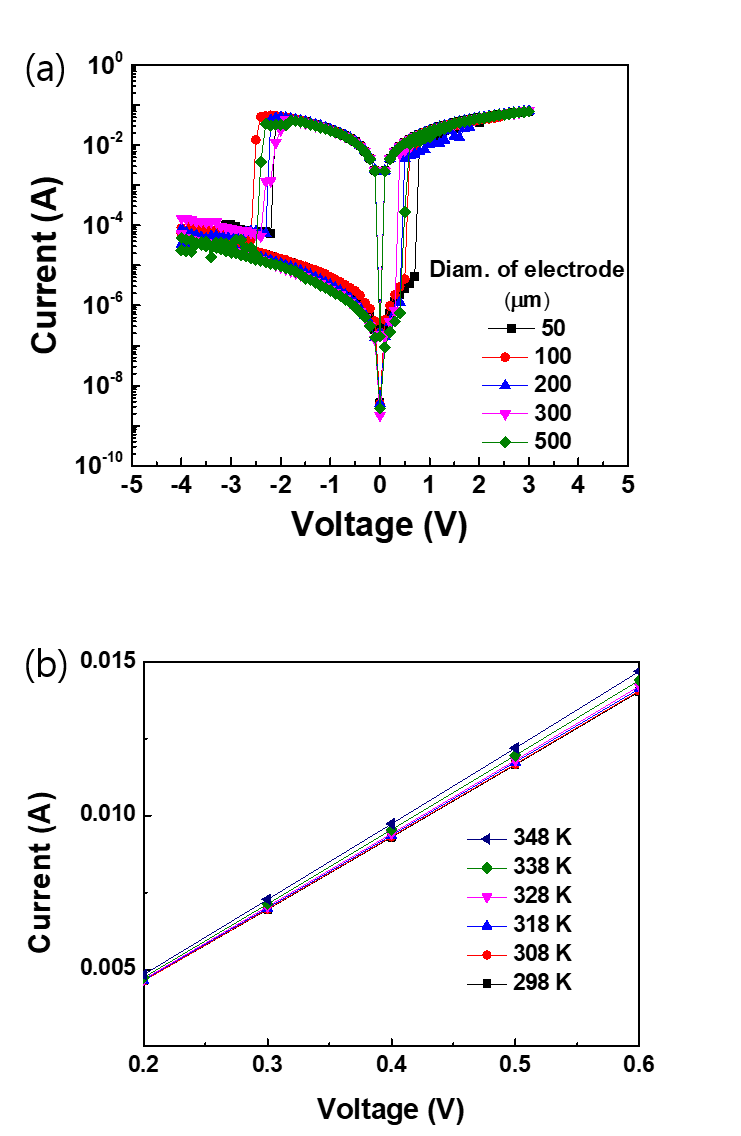


**Figure S5.** (a) I-V curves for different electrode sizes from 50 μm to 500 μm. (b) I-V characteristics in the LRS at temperatures from 298 K to 348 K. The current in the LRS is seen to increase with increasing temperature.

**Figure S6.** Standard deviations for potentiation and depression according to the number of training.


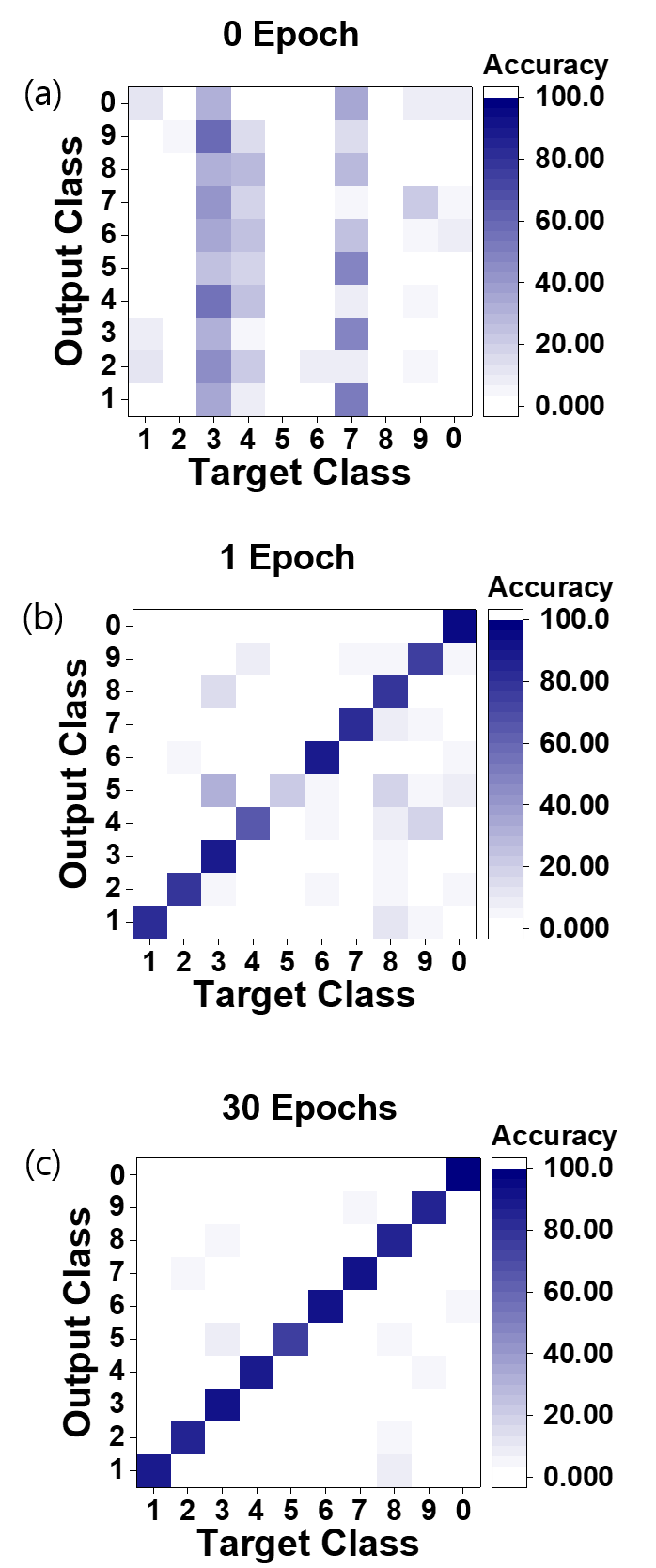


**Figure S7.** Digit-classification results for the learning and the inference test at (a) 0 epoch, (b) 1 epoch, and (c) 30 epochs. The confusion matrices (10×10) between the target classes (input digit) and the output classes (learning phases).

**MNIST simulation**

**Fig. S8** shows the overall simulation as a simple flowchart. To calculate the weights to be mapped to the synaptic device, we used the MatlabR2018b tool to simulate a neural network. The neural network consists of an input layer with 784 neurons and an output layer with 10 neurons. The input and the output neurons are fully connected through 7,840 synapses. The rectified linear unit (ReLU) function for the activation function, the cross-entropy loss function for the error function, and the softmax function for processing the output signal are used. Each function is calculated as follows:

ReLU function: $y=\left\{ \begin{aligned} x, &x\geq0 \\ 0, &x<0 \end{aligned} \right.$ ,

Softmax function: ${p(y)}_{j}=\frac{e^{y_{j}}}{\sum_{k=1}^{K} e^{y_{k}}} for j=1, \cdots, K$ ,

Cross-Entropy loss function: $CE=-\sum_{i=1}^{10} t_{i}\log(p_{i})$ .

Here, $x$ is the sum of the input value multiplied by the weight of the synapse. MNIST patterns, 60,000 with 28$\times$28 pixels, are applied as input values through input neurons. We update the weight using gradient descent back-propagation as a learning algorithm. After training for 30 epochs with this condition, the recognition rate of 10,000 MNIST inference patterns was 91.8% when the learning rate was 0.0002. The trained weight value is normalized and quantized according to a conductance behavior characteristic, such as the ratio of $G_{max}$/$G_{min}$, linearity, and multi-level status, and is mapped to the synaptic device. Here, two types of synaptic devices are required, an excitatory device ($G_{i,j}^{+}$) and an inhibitory device ($G_{i,j}^{-}$), to represent positive and negative weight values. Therefore, one synapse unit is composed of each type of device, and the weight value is $G_{i,j}^{+}$+$G_{i,j}^{-}$. For hardware implementations, integrate-and-firing (I&F) neurons based on capacitors are modeled. MNIST inference patterns are coded at a right-justified rate according to the grayscale and applied to the neural network for inference, as shown in **Fig. S9**.


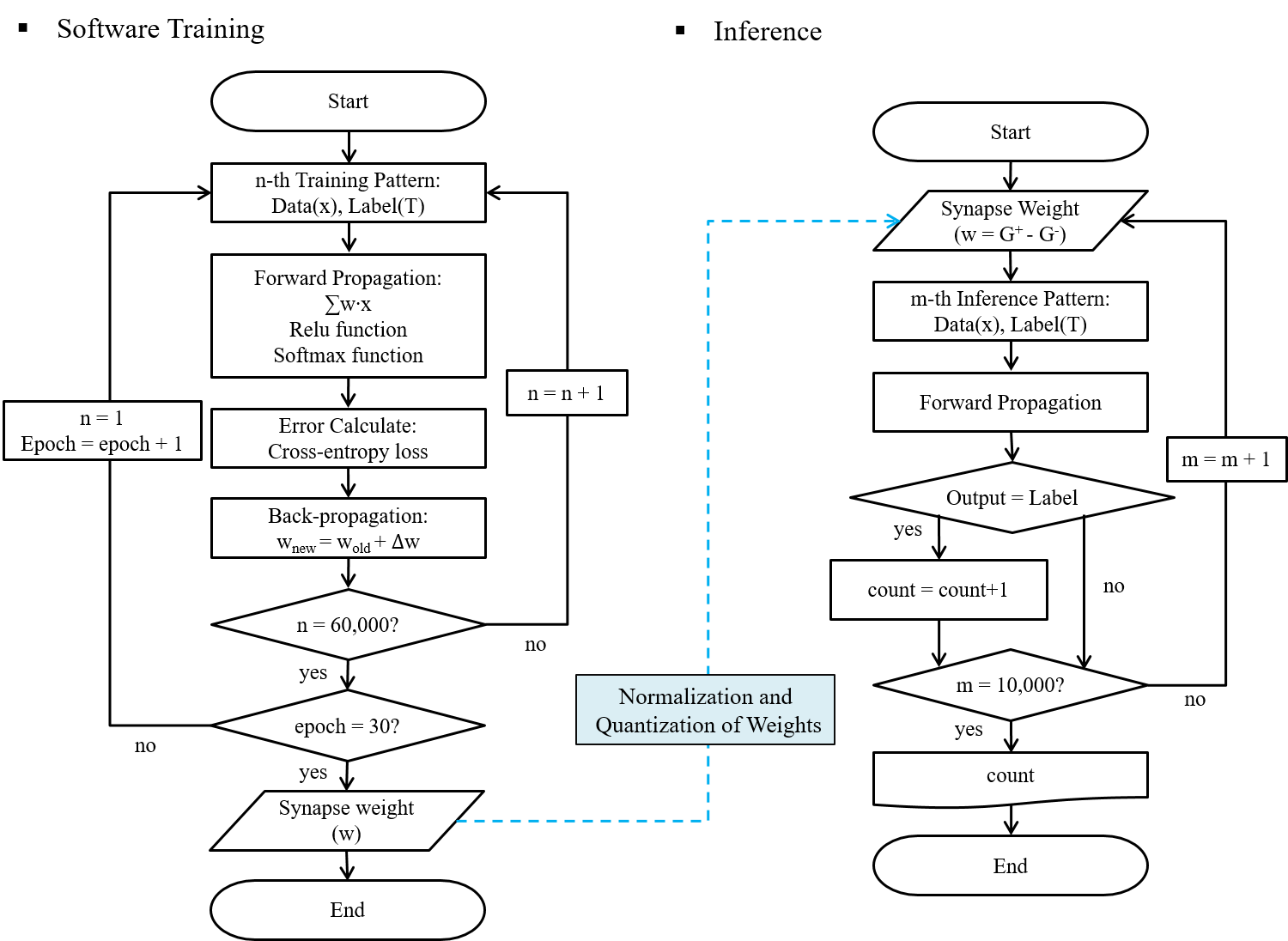


**Figure S8.** Flowchart for the learning and inference process.


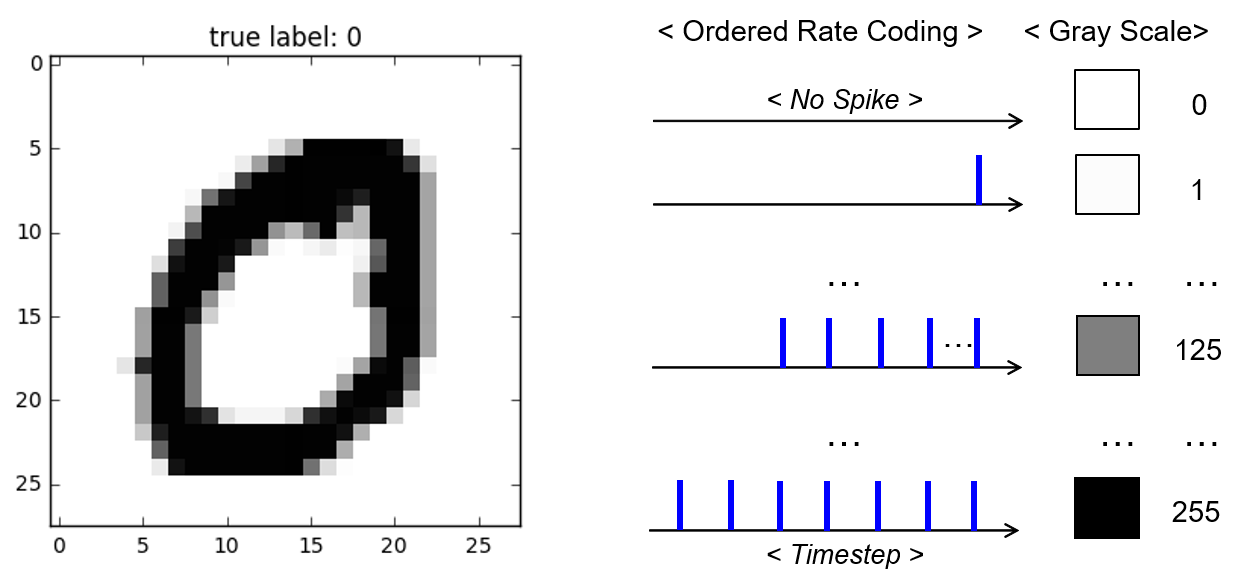


**Figure S9.** Schematic illustration of the righted-justified rate coding in MINST simulation.
